# Structural complexity in ramp-compressed sodium to 480 GPa

**DOI:** 10.1038/s41467-022-29813-4

**Published:** 2022-05-09

**Authors:** Danae N. Polsin, Amy Lazicki, Xuchen Gong, Stephen J. Burns, Federica Coppari, Linda E. Hansen, Brian J. Henderson, Margaret F. Huff, Malcolm I. McMahon, Marius Millot, Reetam Paul, Raymond F. Smith, Jon H. Eggert, Gilbert W. Collins, J. Ryan Rygg

**Affiliations:** 1 University of Rochester Laboratory for Laser Energetics, Rochester, NY USA; 2 Department of Mechanical Engineering, University of Rochester, Rochester, NY USA; 3 Lawrence Livermore National Laboratory, Livermore, CA USA; 4 Department of Physics and Astronomy, University of Rochester, Rochester, NY USA; 5 SUPA, School of Physics and Astronomy, and Centre for Science at Extreme Conditions, The University of Edinburgh, Edinburgh, UK

**Keywords:** Phase transitions and critical phenomena, Structure of solids and liquids, Electronic properties and materials

## Abstract

The properties of all materials at one atmosphere of pressure are controlled by the configurations of their valence electrons. At extreme pressures, neighboring atoms approach so close that core-electron orbitals overlap, and theory predicts the emergence of unusual quantum behavior. We ramp-compress monovalent elemental sodium, a prototypical metal at ambient conditions, to nearly 500 GPa (5 million atmospheres). The 7-fold increase of density brings the interatomic distance to 1.74 Å well within the initial 2.03 Å of the Na^+^ ionic diameter, and squeezes the valence electrons into the interstitial voids suggesting the formation of an electride phase. The laser-driven compression results in pressure-driven melting and recrystallization in a billionth of a second. In situ x-ray diffraction reveals a series of unexpected phase transitions upon recrystallization, and optical reflectivity measurements show a precipitous decrease throughout the liquid and solid phases, where the liquid is predicted to have electronic localization. These data reveal the presence of a rich, temperature-driven polymorphism where core electron overlap is thought to stabilize the formation of peculiar electride states.

## Abstract (web summary)

The properties of materials can be drastically modified under extreme pressure. Here the authors investigate ramp-compressed sodium to 5 million atmospheres with in situ X-ray diffraction and optical reflectivity, revealing a complex temperature-driven polymorphism and suggesting the formation of a previously predicted electride phase.

## Introduction

The formation of high-pressure electrides (HPE’s) was historically counterintuitive for several reasons. First, it was expected that with increasing pressure, atoms seek the most densely packed organization, but HPE’s require the existence of empty sites for electron localization and an implicit volume increase. Second, the Thomas–Fermi statistical model together with Mott’s^1^ or other pressure ionization models (e.g., Stewart–Pyatt^2^) suggest that materials become better conductors with increasing pressure, but HPE’s can have the majority of their valence electrons—unbound at ambient conditions—trapped in the interstices at extreme pressure. Electron localization at high pressure has been predicted at 0 K for many materials and some experimental evidence has been found at room temperature^3^. The degree to which electron localization influences the high-temperature solid and fluid order remains an outstanding question, where thermal effects have a tendency to broaden electron energy bands and modify ionic interactions and electron–phonon coupling. Recent theoretical works predict that the electride character persists up to and into the fluid phase for Na^4^, Li^5^, and K^6^.

**Fig. 1:**
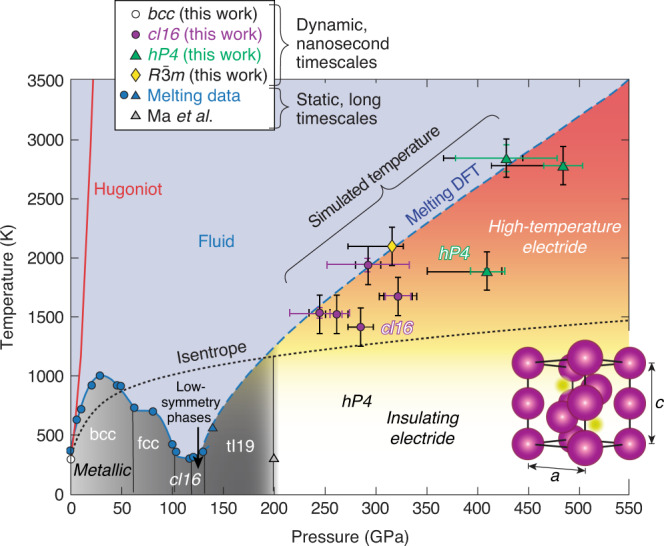
High-pressure Na phase diagram. The high-pressure phase diagram of Na based on our laser-driven ramp-compression data (black error bars represent systematic and random uncertainties; color “error” bars represent standard deviation in pressure and temperature states within the sample) and previous work from refs. ^3,4,21,22,32^. The data are compared to the theoretical principal Hugoniot and isentrope. The melting curve data from refs. ^22,21^ are shown along with a Kechin^33^ fit to density-functional-theory (DFT) calculations^4^ for the melting curve (blue dashed line) above 130 GPa in the *hP4* phase (structural model (bottom-right): Na^+^ ions (purple), localized electrons (yellow)). Four different phases of Na, bcc, *cI16*, $R\bar{3}m$ and *hP4*, are observed. The temperatures are estimated from hydrodynamics simulations (see Supplementary Discussion). The present reflectivity and X-ray diffraction data along with computations suggest a high-temperature electride region (shaded orange).

In high-pressure alkali metals, the forces driving the valence electrons into the interstices are responsible for a cascade of structural and electronic changes in the solid and fluid phases and dramatic evolution of the melting temperature with increasing pressure (Fig. 1). In the case of Na, the melting temperature increases normally up to 30 GPa and 1000 K and then drops all the way down to room temperature by 100 GPa before increasing again. This is particularly interesting because Na has no solid–solid transformation at the 30 GPa inflection point, suggesting that there must be a phase transformation in the liquid to cause the change in relative densities. Experimentally, it is exceptionally difficult to study high-temperature alkali metals, which tend to destroy diamonds in traditional diamond-anvil-cell compression experiments. Many theoretical studies have attempted to explain the remarkable pressure evolution of the melting temperature, some claiming transformation in the liquid to a local order more characteristic of the higher-pressure face-centered-cubic (fcc) and *cI16* phases^7^, and some argue for more subtle changes in local order^8^ or band structure effects^9^.

Here, we report on laser-driven measurements suggesting an electride phase. Laser-driven experiments allow access to unprecedented pressures (484 GPa) and temperatures (~3000 K) but require a nearly isentropic thermodynamic pathway resulting in pressure-driven melting and recrystallization on nanosecond time scales due to sodium’s unusual melting curve. Although 0-K calculations predict that the *hP4* phase is stable from 0.2 to 1.75 TPa^10^, a series of phase transitions were observed upon recrystallization with the *hP4* phase only appearing at the highest compressions (above 400 GPa). We observe the *cI16* phase forming from the liquid, a potential confirmation of the prediction that the liquid has transformed to *cI16*-like local order^7^. Simultaneous reflectivity measurements show a precipitous decrease throughout the liquid and solid phases and are consistent with theoretical predictions that electron localization persists into the liquid phase in the form of dynamic electrons bubbles in a Na^+^ ionic fluid^4^. We present experimental evidence suggesting that electron localization continues to have an important impact on atomic order in the presence of significant thermal effects.

## Results and discussion

**Fig. 2:**
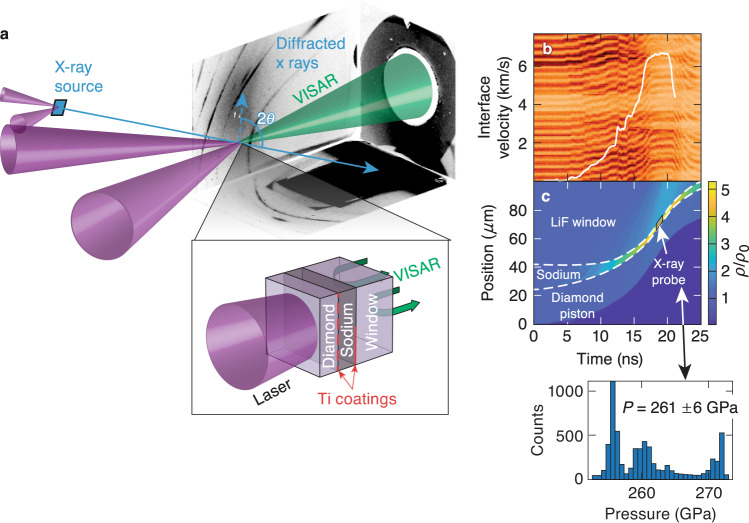
Experimental configuration. **a** The experimental setup for simultaneous X-ray diffraction and reflectivity measurements using LiF or MgO windows. **b** Example velocimetry data and the corresponding Na–LiF interface velocity (shot 27967). **c** A simulated space-time map of the compression, *ρ*/*ρ*_0_, and the pressure histogram in the Na sample during the X-ray probe (shot 27967).

**Fig. 3:**
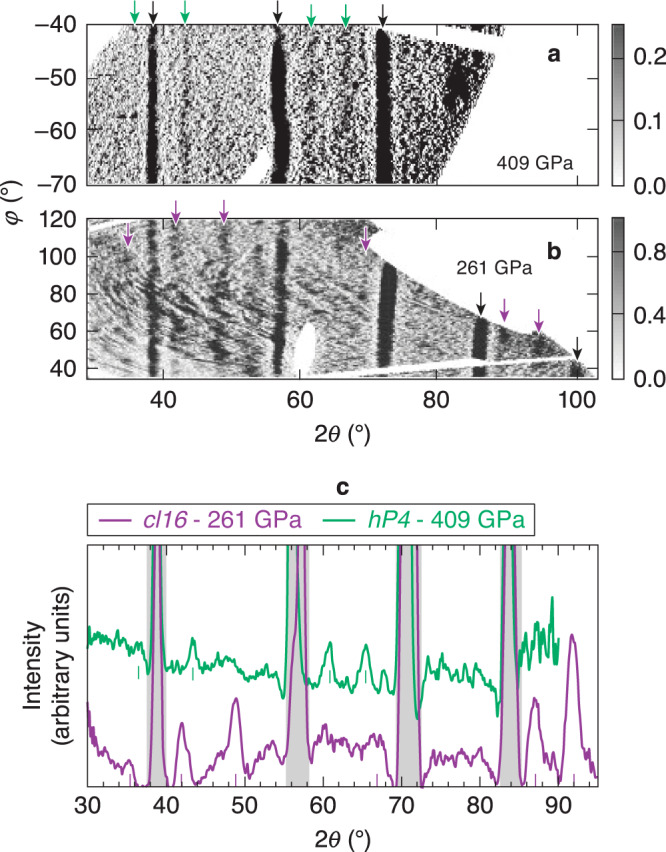
X-ray diffraction data. **a**, **b** Diffraction data at 409 ± 15 GPa (shot 25877) and 261 ± 11 GPa (shot 27967), respectively. The Debye–Scherrer rings from compressed Na are marked with green and purple arrows. Black arrows mark W calibration diffraction peaks. **c** Lineouts along 2*θ* for the image-plate data in (**a**) and (**b**) for *cI16* (purple curve) and *hP4* (green curve).

Previous studies on high-pressure Na were all performed under static compression at or below 1000 K temperature. To explore the high-pressure, high-temperature solid-state structure and reflectivity of Na above 200 GPa, we performed laser-driven compression experiments on the OMEGA EP laser^11^ to measure the crystal structure, pressure, and 532-nm reflectivity of ramp-compressed Na using XRD and velocimetry. The targets comprise Na samples sandwiched between single-crystal ablators and windows (see Fig. 2). A striped Ti coating is applied to the ablator-Na interface to detect Na transparency because the pattern would be imaged using line-imaging velocimetry if Na became transparent. Na was quasi-isentropically compressed, along a path similar to the dashed-black line in Fig. 1, to pressures up to 480 GPa, accessing thermodynamic states bounded by the principal Hugoniot (locus of states accessible by a single shock wave) and the principal isentrope. The Na first melts from the bcc phase, below ~50 GPa, before recrystallizing at higher pressures as it crosses the melting curve again above 200 GPa. Figure 3a–c show examples of the XRD data that are collected using the Powder X-ray Diffraction Image Plate (PXRDIP) platform^12^, depicted in Fig. 2a. High intensity 1-ns-long laser pulses on secondary targets generated a pulsed, quasi-monochromatic 8.37 keV X-ray source (XRS) that is collimated by a pinhole placed behind the sample (see Methods). The Debye–Scherrer rings from the compressed Na sample are collected in a box lined with image plates (IPs)^13^. On each shot, the spatially- and temporally-resolved velocity and reflectivity (for targets with transparent windows) of the target are recorded with a line-imaging velocity interferometer system for any reflector (VISAR)^14^ (Fig. 2b). The pressure of the Na sample at the XRD probe time is inferred using the velocity histories measured with VISAR fitted by radiation-hydrodynamic simulations (Fig. 2c). The intensity of the VISAR signal provides information about the target reflectivity. Because no direct temperature measurements are made, they are estimated using the hydrodynamic simulations^15^ (see Supplementary Discussion). Using streaked optical pyrometry measurements, an experimental upper bound of 3500–4300 K on the Na temperature is inferred assuming the LiF window is neither absorbing nor emitting over the 590–700 nm diagnostic wavelengths^16^.

**Fig. 4:**
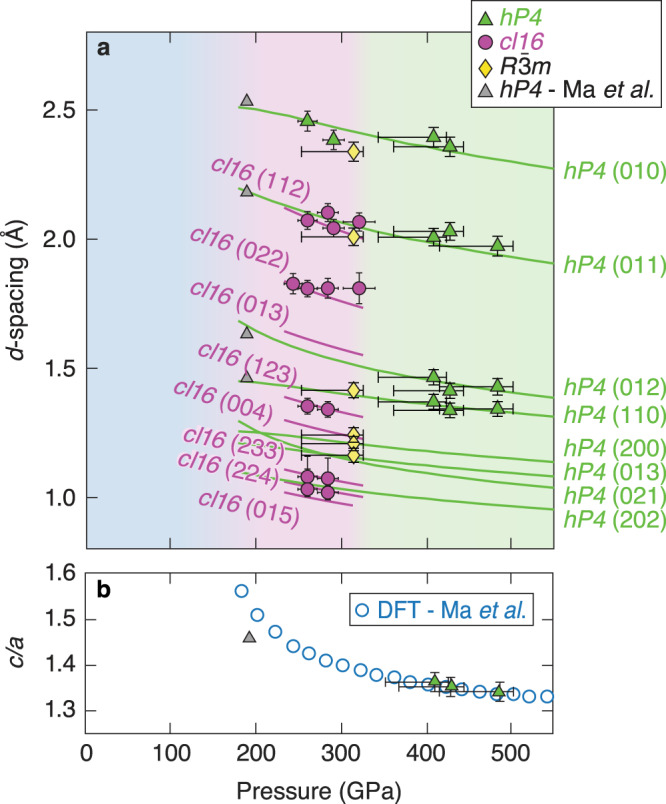
Data compared to theoretical predictions. **a** A comparison of the measured *d*-spacing versus pressure from this work, 0 K DFT equation of state assuming the *hP4* structure^3^ (green curves), a Vinet fit to the *cI16* data (purple curves)^34^, and static compression experiments^3^. Error bars defined in Supplementary Discussion. **b** Changes in the *c/a* axial ratio in the *hP4* phase are in excellent agreement with 0 K DFT predictions^3^.

A diffraction pattern from Na compressed to 409 ± 15 GPa is shown in Fig. 3a. The diffraction pattern has four peaks from compressed Na that is consistent with the *hP4* phase reported in previous room-temperature static compression experiments by Ma et al.^3^ but at higher temperatures and under dynamic compression. The *hP4* phase is a distorted double hexagonal close-packed (*dhcp*) lattice of Na ions that, together with the localized electrons, form the binary Ni_2_In-type structure (Fig. 1). We index the four lines as the *hP4* (010), (011), (012), and (110) giving lattice parameters *a* = *b* = 2.75 ± 0.03 Å and *c/a* = 1.35 ± 0.02. Compared to the ideal *dhcp* lattice, with *c/a* = 3.27 for hard spheres, the *hP4* structure is highly distorted and not close-packed because the electride nature of this structure stabilizes a more open structure to accommodate the localized valence electron charge^3^. Compared to the static compression data (*c/a* = 1.46), we observe a decrease in the *c/a* ratio with increasing pressure (Fig. 4b) that agrees well with the DFT predictions and implies a stronger electron localization^3^.

At pressures between 242 and 292 GPa, the XRD pattern is consistent with a complex *cI16* phase. This is isostructural with the *cI16* phase of sodium near the minimum of the melting curve at lower temperatures and pressures (108 GPa, room temperature)^17^ and lithium (40 GPa, 180 K)^18^. The *cI16* structure (space group: $I\bar{4}3d$, 220) is a bcc superstructure with sixteen atoms on the *16c* Wyckoff site. Figure 3b shows an example diffraction pattern at 261 ± 11 GPa. The diffraction pattern has five peaks with a clearly different symmetry than the *hP4* structure, which was observed at these pressures in room-temperature experiments^3^. A comparison of the two integrated XRD patterns is shown in Fig. 3c.

In one experiment at 315 ± 11 GPa (shot 26479), a diffraction pattern distinct from both *cI16* and *hP4* is observed. The diffraction pattern was compared to those from theoretically predicted structures and other structures observed in alkali metals including *oP8*, *oC16*, *tI4*, and *tI19* but none were found to match the observed diffraction pattern. A Bravais lattice and space group search^19^ suggest a rhombohedral structure (space group: $R\bar{3}m$, 166) with lattice parameters, *a* = *b* = 2.85 ± 0.03 Å and *c* = 6.02 ± 0.06 Å (hexagonal axes), and a density of 5.40 ± 0.2 g/cc. This is the same structure observed in As, Sb, and Bi^20^. Although the proposed *cI16* and $R\bar{3}m$ structures are consistent with the data, the number of observed peaks is not sufficient to uniquely identify the phases. Structure search calculations find a number of energetically competitive and dynamically stable structures in this regime at finite temperatures. This includes phases with ${R}3,{P}{2}_{1}/{m},{C}2/{m},{P}{6}_{3}/{m},{P}\bar{3}{m}1,{Fmm}2,{P}\bar{6}2{m}$ spacegroups (E. Zurek, personal communication, December 7, 2021). Due to preferred orientation and limitations in instrument sensitivity, the structure solution may be even more complex than the structures suggested here. Additionally, the two new phases of Na observed here may represent non-equilibrium phases that are stabilized by the rapid, nanosecond melting and recrystallization inherent in these laser experiments. Theoretical investigation of the kinetic stabilization of these phases is needed.

The *d*-spacings deduced from the XRD data versus the Na pressure inferred from VISAR for the three different phases are shown in Fig. 4a. The *cI16* peaks that are not observed are obscured by pinhole calibration peaks. In two experiments, a low-angle peak consistent with the *hP4* (010) peak is observed along with data consistent with the *cI16* phase. This can be caused by distribution of pressures within the relatively thick Na sample layer (Fig. 1 purple, green, and yellow “error” bars show standard deviation of pressure and temperature distributions).

Observation of solid Na at 244 to 484 GPa indicates that Na melts and recrystallizes on nanosecond time scales and that the melting temperature continues to increase dramatically above 137 GPa, where it is only ~400 K^21,22^. Due to the steep melting curve, the solid is denser than the liquid, and the measurement of the *cI16* structure may suggest the liquid is retaining a local order similar to the lower-pressure phases of Na. This is supported by high-temperature ab initio calculations for molten sodium that report a transition to a lower-coordinated liquid at 65 GPa^7^.

**Fig. 5:**
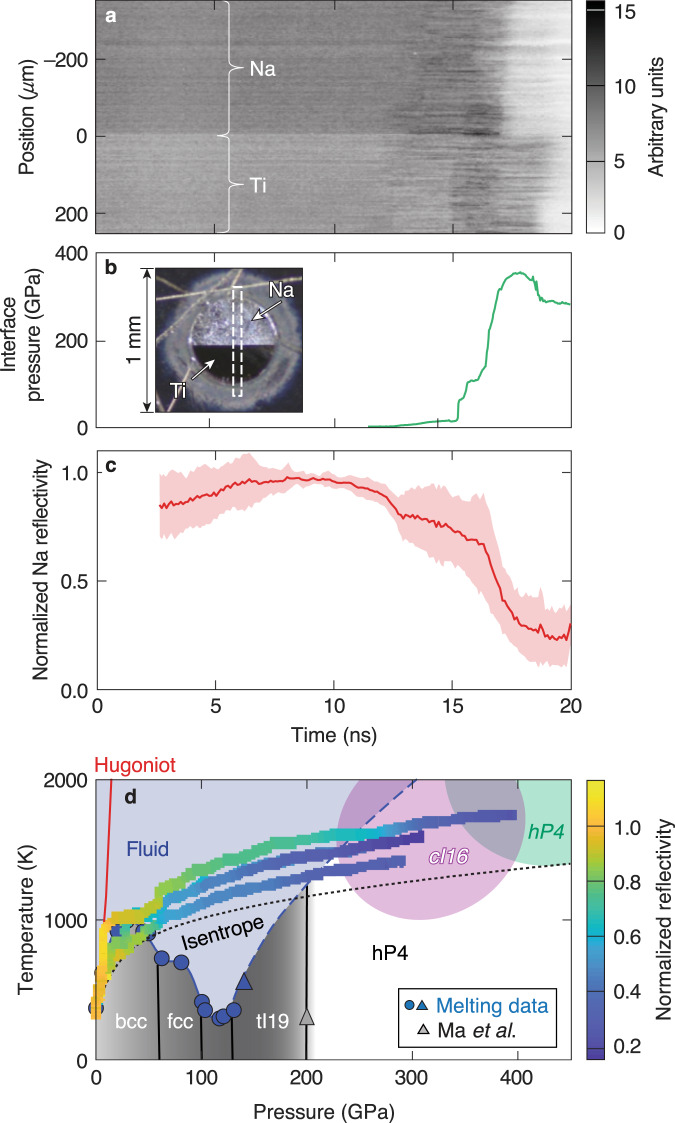
Reflectivity data. **a** A non-fringing VISAR image for a Na target using a transparent MgO window and containing Ti coatings to detect changes in reflectivity (shot 27971). The non-fringing image is generated by blocking one arm of the VISAR interferometer, and shows no evidence of the preimposed striped reflectance pattern with 150 μm period behind the Na layer. **b** (inset) A microscope image through the high-Z pinhole shows the Na layer (top) and the half-Ti overcoat (bottom) with the VISAR field of view overlaid (dashed-box). The interface pressure of the shot shown in (**a**) shows that the drop in reflectivity is coincident with the increasing pressure. **c** Average (red) and standard deviation (red shaded region) of all Na reflectivity data normalized to the Ti reflectivity behind the transparent window. **d** The temperature-pressure phase diagram of Na with the simulated ramp-compression path (multicolor curves) for three reflectivity experiments.

Optical data reveal that the reflectivity of the high-pressure Na drops significantly, but it does not become transparent at the temperatures accessed here. The preimposed reflectance pattern behind the Na is never observed because the Na initially crystallizes into the *cI16* phase instead of *hP4* and indicates that the attenuation length remains below the sample thickness (~3 μm at peak compression) at 532-nm. Example VISAR data is shown in Fig. 5a. Return signal corresponding to the Na–MgO interface is on the top-half of the image while the signal from the Ti–MgO interface is on the bottom-half. At ~17 ns, there is a drop in the reflected signal on the Na-half of the target compared to the Ti-half that coincides with the peak Na pressure.

Figure 5c shows the average Na reflectivity as a function of time. As seen in both the raw data and the average across all experiments, the Na reflectivity drops to about 23 ± 4% of its initial value. The temperature-pressure paths of the reflectivity measurements are shown in Fig. 5d where hydrocode simulations are used to estimate the temperatures. The conditions within the sample are relatively uniform, therefore the stresses probed in the reflectivity measurements at the sample-window interface are representative of the bulk conditions as measured in the XRD. The reflectivity is tracked through the bcc phase into the stability region of the fluid phase, and at the highest pressures, in the *cI16* phase and approaching the *hP4* phase where it is dark and non-reflective. The reflectivity shows a small drop corresponding to the initial shock melting and a more pronounced drop upon recrystallization. A threefold drop in electrical conductivity is expected in the low-coordinated liquid sodium between 40 and 80 GPa^7^. Similarly in liquid potassium, atomistic simulations predict a continuous transition from a free-electron metal to an electride liquid at pressures corresponding to the melting curve maximum and the onset of electride formation (10–20 GPa (K); 30–200 GPa (Na)) that manifests as a dip in the reflectivity similar to that observed here^6^. Reduced reflectance is consistently observed in host–guest structures in Na and K at lower pressure^3,23,24^.

Using laser-driven ramp compression, XRD measurements of sevenfold compressed Na are made in a regime where core overlap is thought to stabilize the formation of electride states. The observation of the *hP4* phase at 480  GPa and ~3000 K that was previously observed to be transparent at 200 GPa and room temperature suggests that electride formation is possible on nanosecond time scales and at higher temperatures. At intermediate pressures, two additional unexpected phases of Na are observed demonstrating the presence of a rich polymorphism. The reflectivity in both the liquid and solid stability regions is observed to continuously decrease where theory predicts a liquid-liquid phase transition to an electride fluid in alkali metals^4,6^. Interactions between core electrons occur in all materials at extreme densities and pressures, and these results give insight into the structural complexity and core-electron chemistry in Na—the most striking example of a high-pressure electride.

## Methods

### Target preparation and design

The targets comprise Na samples (8 to 65 μm thick) sandwiched between single-crystal diamond ablators and single-crystal diamond, LiF, or MgO windows. The rear diamond windows become opaque to the velocimetry probe beam as it is ramp-compressed, whereas the LiF and MgO windows remain transparent^25–27^. Accordingly, targets with LiF and MgO are used for simultaneous XRD and reflectivity measurements, while the diamond window targets are used for XRD-only experiments. A striped Ti coating is applied to the diamond(ablator)–Na interface (dashed red line Fig. 2a) that would be imaged on the top-half of the VISAR data if Na became transparent. A half-coating of Ti is applied to the Na–LiF interface (solid red line Fig. 2a) to calibrate the Na reflectivity measurement and determine the transmission of the LiF window. The targets were assembled in a ultra high-purity glove box with either an Ar or N_2_ environment. No glue was allowed between the Na sample and the window; this is necessary for reflectivity measurements on the Na sample without knowledge of the glue transmission and absorption. The reflectivity of the Na was inspected for oxidation before the experiments by looking for evidence of darkening and dulling of the sample using a reflectance microscope. The targets were transported directly from the glove box to the target chamber to minimize risk of Na degradation. Additional details about the target preparation are discussed in the Supplementary Methods.

### In situ X-ray diffraction

Two beams of the Omega EP laser ramp compressed the Na target for 20-ns and two additional beams irradiated a Cu foil with 1-ns pulses to create 8.37 keV X rays for XRD^28^. The x rays are generated over the duration of the pulse length and near the end of the 20-ns compression pulse when the Na is up to seven-fold compressed and at a peak, uniform pressure. The X rays are collimated by a W or Ta pinhole aperture located behind the Na sample and collected in an IP-lined box. In addition to diffraction from high-pressure Na, XRD patterns from the edges of the uncompressed W or Ta collimating pinhole (Fig. 3a–c) black arrows and gray shaded regions] are evident, and are used to determine the image plate, XRS, and pinhole locations relative to the diffraction lines^29^. The high-Z pinhole limits the field of view of the IPs to the center of the 1100 μm-diameter driven region of the target and provides additional shielding of the IP’s from the large X-ray background from the XRS and Na target ablation plasma.

Diffraction from the Na was distinguished from other background features using the expected characteristic peak width (0.37 degrees in 2*θ* for the smallest pinholes) calculated from the instrumental broadening in the absence of pinhole broadening. Features, wider or narrower, were not included in the structure search. The intense diffraction peaks are powder-like, therefore features that had limited azimuthal extent were also not included. The exclusion of these features did not reject structures with a diffraction peak in that location, therefore this conservative approach was used in the data analysis.

### Reflectivity

The background-subtracted charge-coupled-device counts in the VISAR image corresponding to the Na–window interface and the Ti–window interface are normalized to the Ti–window side. The normalization accounts for the overall decrease in the VISAR return signal with time due to the increasing optical absorption of the window as the compression wave propagates^30^. The change in the MgO and LiF window refractive indices during ramp compression are taken into account using those measured by refs. ^27,26^, respectively. Although the initial reflectivities were as expected under diffuse white light (see microscope image in Fig. 5), the expected initial signal ratio of Na/Ti ( ~ 2.0) as measured by the coherent VISAR source was reduced and varied between targets. This is due to diffuse scattering from large-scale structures in the sodium sample causing reflections outside the ±9^∘^ acceptance angle of the *f*/3.3 VISAR collection optic compared to the specular Ti coating. An average Na/Ti ratio of 1.1 ± 0.5 is measured with VISAR.

The reflectivities are calculated assuming a fixed value for the Ti refractive index. The reflectivity is normalized to the vaccuum reflectivity (96.2%) for times of maximum reflectivity. The Na reflectivity first increases slightly before dramatically decreasing at late times. Complete loss of signal at ~19 ns is attributed to shock formation in the MgO window as detected by the time-resolved streaked optical pyrometer^16^.

Due to a strongly anisotropic absorption spectrum^31^, Na is predicted to be optically transparent for light polarized in the *a–b* plane but reflective for light polarized along the *c* axis in the *hP4* phase. At 420 GPa, it is predicted the optical depths at the 532-nm VISAR probe wavelength are 1200 and 5 μm for the in-plane and *c*-axis polarizations, respectively, using dielectric constants from ref. ^31^.

## Supplementary information

Supplementary Information

## Data Availability

The source data shown in the figures are provided in Supplementary Tables I, II. All the other data supporting the findings of this study are available from the corresponding author upon request.

## Code availability

There are no custom codes or mathematical algorithms central to the conclusions here. The publicly available GSAS-II code can be found here https://subversion.xray.aps.anl.gov/trac/pyGSAS.
