# Supplementary material for: Structural complexity in ramp-compressed sodium to 480 GPa
Source: Nat Commun. 2022 May 9;13:2534. doi: 10.1038/s41467-022-29813-4 (PMC9085792; doi:10.1038/s41467-022-29813-4)
Supplement: Supplementary file 1 — Supplementary Information [file 41467_2022_29813_MOESM1_ESM.pdf]

Supplementary Information: Structural Complexity in Ramp-Compressed Sodium to 480 GPa

D. Polsin *et al.*

I. SUPPLEMENTARY METHODS

A. Target preparation

Due to Na's reaction with water, the targets were assembled in a ultra-high-purity glove box with either an Ar or N_2 environment. The targets comprise Na samples (8 – 65 μm thick) sandwiched between $\langle 110 \rangle$-oriented ∽30-μm single-crystal diamond ablators and 67–103 μm thick $\langle 100 \rangle$-oriented single-crystal LiF, $\langle 100 \rangle$-oriented MgO, or $\langle 110 \rangle$-oriented diamond windows. The Na metal was thinly sliced and then compressed between the diamond ablator and window to the desired thickness using a piston cylinder cell. No epoxy is applied between the Na and window to eliminate the need to include the epoxy transmission in the reflectivity determination. Prior to removal from the glove box, the target is sealed with a continuous layer of epoxy around the target perimeter to prevent reaction with the atmosphere during transportation from the glove box to the target chamber.

Titanium coatings on the target ablator and rear window allow for a measure of the change in Na reflectivity as it is compressed. A Ti coating (1000 Å) on the bottom-half of the transparent LiF window and Ti stripes (1000 Å) on the diamond ablator are in contact with the Na. Ti is chosen to maintain high dynamic range in the reflectivity sensitivity because of its reflectivity at 532 nm (57.5 %). The VISAR probe beam reflects off the Ti coating through the transparent window providing unambiguous tracking of the Na-window interface velocity throughout the compression of the Na.

B. *In situ* x-ray diffraction

In these experiments, the Omega EP lasers compressed Na to pressures reaching 480 GPa using a shock-ramp compression technique. To prevent the ramp compression wave from steepening into a shock wave, two 10-ns pulses were stitched together to create a 20-ns ramped laser pulse shape with a low-energy "foot" that pre-compresses the Na before the second pulse compresses to the final maximum pressure as shown in SI Figs. 1 and 2. Two beams irradiated the Na sandwich target at an angle of 23° with respect to the target normal with a peak irradiance of 3.4 $\times 10^{12}$ W/cm^2. These drive beams used distributed phase plates that produced a focal spot with a super-Gaussian intensity distribution $\{I = I_0 \times \exp\left[-2\left(r/550\mu\text{m}\right)^{8.0}\right]\}$ to ensure transverse spatial uniformity in the sample. Two additional 1250-J beams irradiated a Cu foil with 1-ns square pulses at 2.5 $\times 10^{15}$ W/cm^2 to create 8.37 keV He-α x-rays for XRD [1]. The x rays are generated over the duration of the pulse length (1-ns) and near the end of the 20-ns main drive.

The experiment uses the Omega EP x-ray diffraction platform, Powder X-ray Diffraction Image Plates (PXRDIP), that is described in Refs. [2, 3]. The Debye-Scherrer rings from the compressed Na sample are collected in an image plate (IP) lined box with Kapton (25 μm) and Cu (12.5 – 25 μm) filtering to attenuate background x rays other than Cu He-α. IP data analysis involves mapping IP pixels onto the scattering angle, 2θ, according to Ref. [2]. The lattice plane d spacings are calculated using the Bragg condition, $\lambda = 2d\sin(\theta)$, for an x-ray wavelength, λ, and x-ray incidence angle, θ. The 2θ resolution for each peak is about 1 degree for a Cu x-ray source (XRS) considering spectral broadening, finite XRS size, and a finite pinhole diameter [3]. In addition to diffraction from Na, calibration diffraction patterns from the edges of a 300-μm or 800-μm diameter W or Ta pinhole (75-μm thick) are used to determine the IP, XRS, and pinhole locations relative to the diffraction lines. The larger pinhole diameter is used for the reflectivity measurements to observe the spatial features on the target (Ti stripes). A 300-μm diameter pinhole is used for the XRD-only experiments to reduce the effects of pinhole broadening and transverse pressure gradients.

Because the pinhole diameter (800-μm) is larger than typically used (300-400-μm) and similar to the laser drive spot diameter (1100-μm), the contribution of transverse pressure gradients to the pressure distribution at the time of x-ray exposure is investigated. The transverse-pressure uniformity across the 800-μm line-imaging VISAR field-of-view is evident from the planarity of the fringes as shown in Supplementary Figure 3 (a) for shot 27967. For this shot, the pressure of the *cI16* Na sample is 261 $\pm$ 11 GPa. For the standard Omega EP PXRDIP geometry as described in Ref. [3] (α = 22.5°, r_x = 24.24 mm), a combined pinhole, sample and window thickness of 100-μm-thick at the time of the x-ray exposure, and an 800-μm-diameter, 75-μm thick Ta pinhole, the impact parameter b is calculated. As shown in Supplementary Figure 3 (b), the impact parameter is the distance away from the aperture axis where b_{max} is the maximum impact parameter visible by any given detector element and b_{center} is the impact parameter for a ray that passes through the center of the pinhole axis to a detector element. The 550-μm (radius of laser drive spot) contours of b_{center} (solid blue) and b_{max} (dashed-blue) are plotted on top of *cI16* diffraction data for the same shot. The detector elements to the left of the blue-dashed curve have a b_{max} less than the laser drive spot radius and therefore transverse pressure gradients do not contribute to the pressure histogram in that region. Although b_{max} is larger than the radius of the laser drive spot for the detector elements where two high-angle diffraction lines are observed, b_{center} is less than 550-μm at those scattering angles and better describes the majority of the sample volume contributing to the diffraction. The contribution to the pressure distribution from transverse pressure gradients is taken into account by including an asymmetric

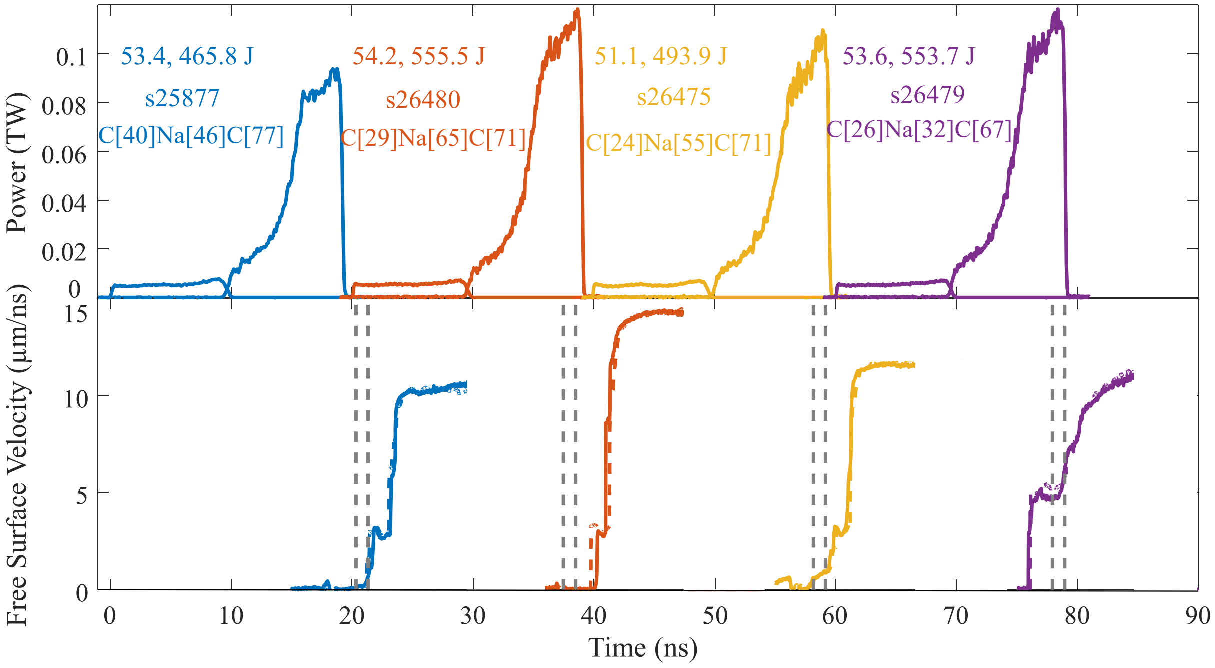

Supplementary Figure 1. **Pulse shapes and free-surface velocities.** Pulse shapes (top) and the free-surface velocities (bottom) for the x-ray diffraction experiments using diamond windows. The curves are shifted in time for clarity. The timing of the x-ray probe is shown by the vertical gray dashed lines. Target dimensions are also listed with units of thickness in [microns].

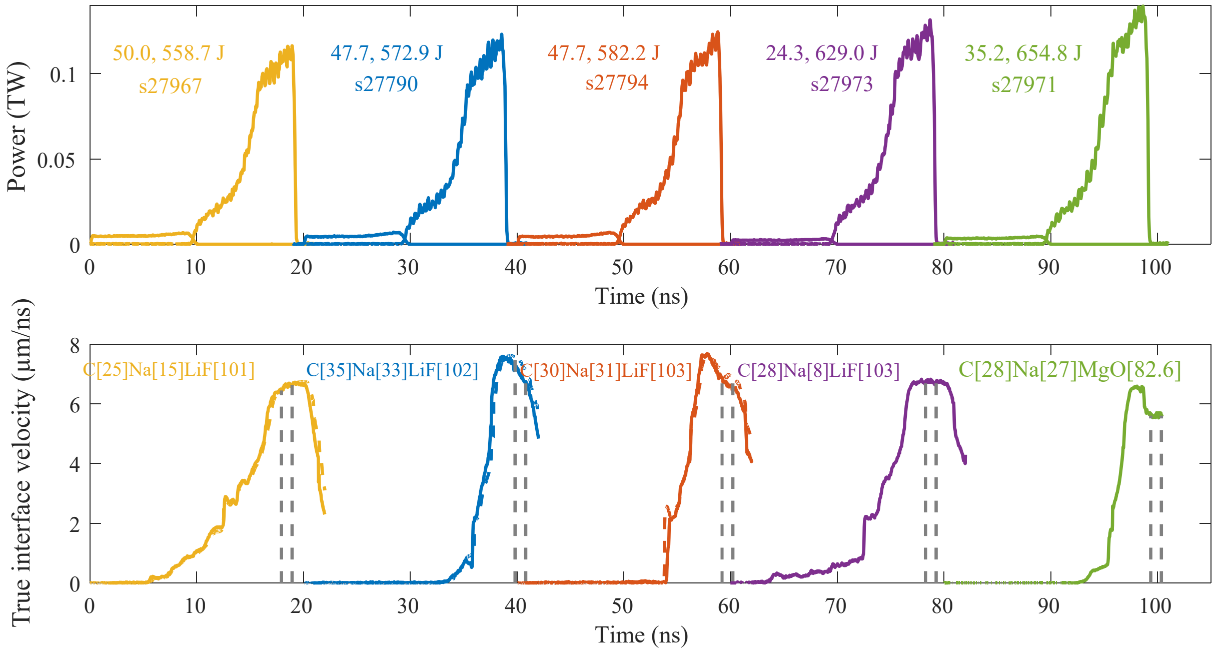

Supplementary Figure 2. **Pulse shapes and interface velocities.** Pulse shapes (top) and the true interface velocities (bottom) for the simultaneous diffraction and reflectivity experiments using LiF or MgO windows. The curves are shifted in time for clarity. The timing of the x-ray probe is shown by the vertical gray dashed lines. The solid and dashed lines for the velocity correspond to velocities extracted from two VISAR interferometers. In some cases, there is only one VISAR measurement because the etalon leg of one interferometer was blocked to collect a non-fringing image. Target dimensions are also listed with units of thickness in [microns].

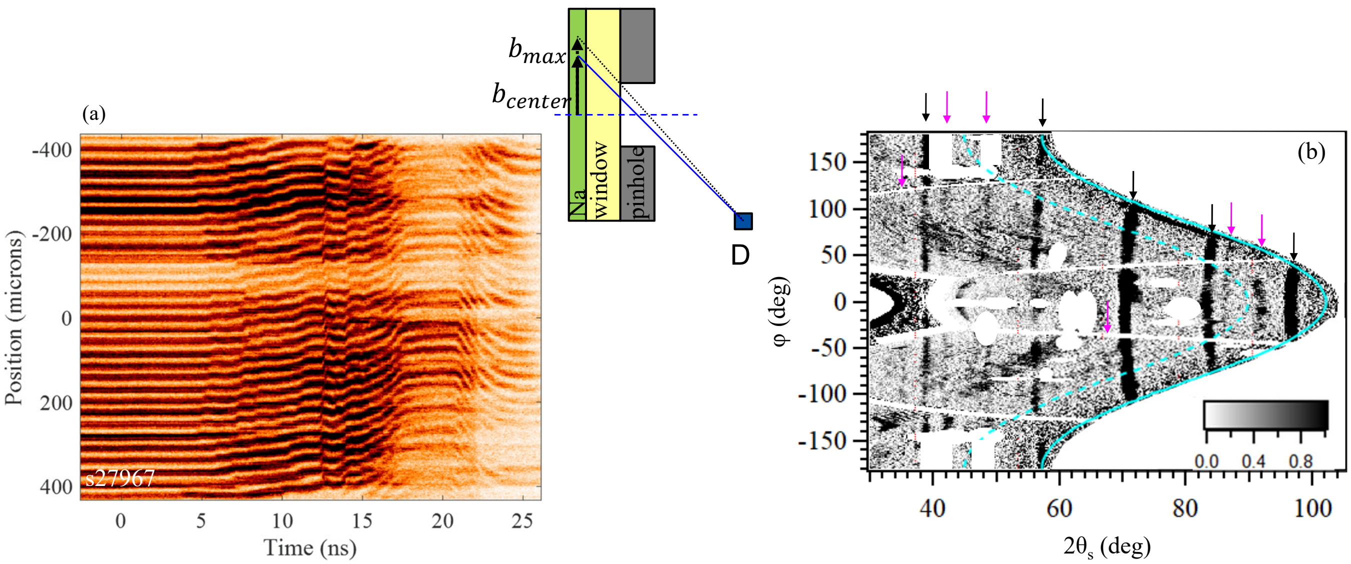

Supplementary Figure 3. **Transverse uniformity.** (a) An example of the line-imaging VISAR data (shot 27967), where the fringe shifts are proportional to the Na-LiF interface velocity. The data shows that both the Na sample layer thickness and the 1100-μm-diameter compression wave are uniform over the 800-μm VISAR field-of-view. (b) PXRDIP IP data from the same shot. Overlaid on the data are the 550-μm (radius of laser drive spot) contours of b_{center} (solid blue) and b_{max} (dashed-blue) for each detector element D showing that the high-pressure volume of the Na sample contributes to the majority of the diffraction data (pink arrows). Black arrows point to calibration diffraction peaks.

d spacing error bar of 0.05 Å for the two high angle diffraction peaks because the range of impact parameters will both broaden the diffraction line and contribute a low-pressure tail to the pressure histogram.

II. SUPPLEMENTARY DISCUSSION

A. Pressure determination

The pressure of the Na sample is deduced using data from a line-imaging VISAR [4] that detects Doppler shifts of a 532-nm probe beam reflecting off a moving surface in the target (e.g. sample interface through a transparent window or free-surface). The Doppler shifts are manifested as shifts in the fringe pattern recorded in a 2-D interferogram. The fringe shifts are proportional to the changes in the velocity of the reflecting surface and these velocities are used to determine the pressure of the Na samples. The intensity of the VISAR signal provides information about the target reflectance. To see the preimposed striped reflectance pattern more clearly, the etalon leg for one of the interferometers was sometimes blocked to collect a non-fringing image.

The pressure in the Na sample at the time of the x-ray exposure is calculated using hydrodynamics simulations shown in Supplementary Figure 4. The hydrodynamics code results are cross-checked with the backwards-characteristics technique [5] and the results are in agreement for experiments that used transparent windows as shown in Supplementary Figure 5 (to within a few percent in the average pressure). Due to different treatments of the diamond strength, the backwards-characteristics technique and the hydrodynamics simulations are not in agreement for experiments that used diamond windows and an additional systematic uncertainty is included. The histogram of pressures within the Na sample during the entire x-ray probe time for a relatively thin (8-μm) and thick (33-μm) sample are shown in Supplementary Figure 6. The standard deviation in the pressure distribution is, as expected, larger for the thicker sample and gives information about the pressure gradients in the sample. The uncertainty in the mean pressure of the sample (black error bars Supplementary Figure 7) is different than this standard deviation (color "error bars" Supplementary Figure 7).

The laser power and Na sample thickness are used as free parameters in the hydrodynamics code, *HYADES* [6], and run iteratively to match the measured interface or free surface velocities by minimizing the χ^2 difference between the measured and simulated velocities. The apparent Na-window interface velocity is first corrected to give the true velocity using the refractive index models for the windows from Refs. [7, 8]. The target layers are defined on a mesh of 1300 zones and EOS models for the diamond, Na, and LiF/MgO are *SESAME* 7834, *SESAME* 2441, and *SESAME* 7271v3/*SESAME* 7460, respectively [9]. The strength of diamond is included using a Steinberg-Guinan model with a shear modulus G_0 80 GPa, yield strength 70 GPa, and pressure-dependence of the shear modulus $A_0 = 1/G_0(dG/dP)$ = 4.3E-3 GPa^{-1} [10]. The converged Na sample thickness used in the simulation is within error of the measured Na layer thickness (10%).

The temperature-pressure phase diagram shown in Supplementary Figure 7 shows the data from this work (pink, yellow, and green points). Two error bars are displayed for both the

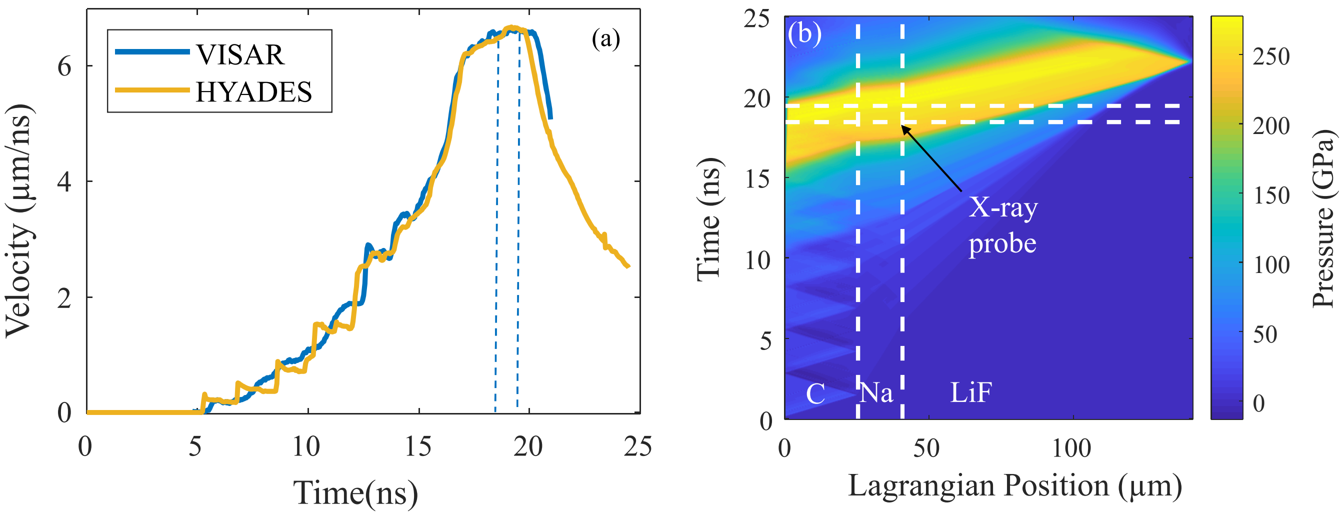

Supplementary Figure 4. **Hydrodynamics simulation optimization.** (a) (shot 27967) The measured interface velocity from VISAR (blue) compared to the hydrodynamics simulation results after optimization (yellow). The x-ray probe (dashed blue lines) is timed when the Na is at a uniform high-pressure state. (b) A pressure map as a function of space and time from the hydrodynamics simulation showing that the Na layer is compressed to 261 GPa at the time of the x-ray exposure (white dashed lines).

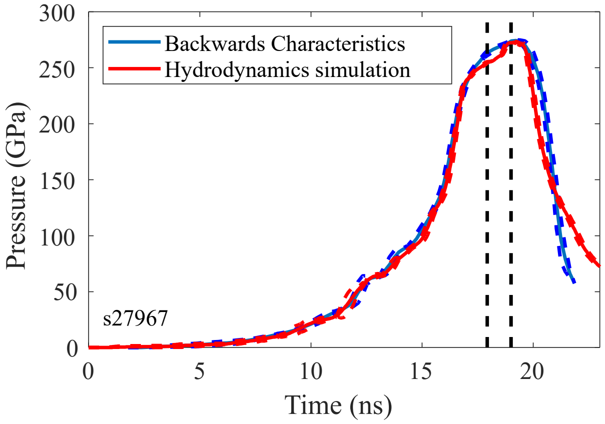

Supplementary Figure 5. **Method of Characteristics.** The average pressure in the sodium sample as a function of time calculated using two independent methods: a backwards characteristics calculation (blue) and HYADES hydrodynamics simulations (red). The dashed lines represent the standard deviation.

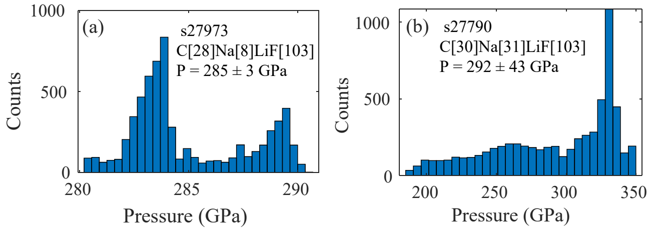

Supplementary Figure 6. **Ensemble of pressures within the sample.** The histogram of pressures within the Na sample during the entire 1-ns x-ray probe time for a relatively thin (a) and thick sample (b).

temperature and pressure. The green, pink, and yellow "error bars" are the standard deviation of the temperature and pressure within the sample at the time of the x-ray probe. The black error bars are the true error bars.

The systematic error that dominates the pressure uncertainty is the mechanical response of the diamond, LiF, and MgO windows. For a relatively thin sample and/or long timescale compression (sample and window pressures have equilibrated), the pressure of the Na sample is independent from the sample EOS but does rely on the window EOS. We have confidence in the window EOS, because the LiF and diamond EOS used in the simulations have been benchmarked with experimental results. The ramp EOS of diamond was measured up to 800 GPa where an error of 3% in CL-Up is reported [11] and contributes a 3% error bar for experiments with a diamond window. Uncertainty in diamond strength is estimated by running hydrodynamics simulation with and without a strength model, leading to an asymmetric error in the resulting pressure of at least 50 GPa [12]. A 3% error is introduced in the pressure determination for targets with LiF windows due to the uncertainty in the ramp EOS up to 350 GPa [13]. A 3% error is included for the experiment that used an MgO window due to the uncertainty in the MgO pressure-density equation-of-state (EOS) measured up to 900 GPa [14]. For LiF and MgO, an additional systematic error of 0.3% and 3%, respectively, is included for the uncertainty in the refractive index correction [7, 8]. All experiments include a VISAR phase extraction uncertainty of 5% of the velocity per fringe (VPF) and the VPF's used in the two interferometers were 2.74 and 1.64 μm/ns/fringe.

These experiments rely on hydrocode simulations to estimate the temperatures because there are no temperature measurements. If a shock-ramp path is assumed, then the small initial shock is expected to be similar in all experiments be-

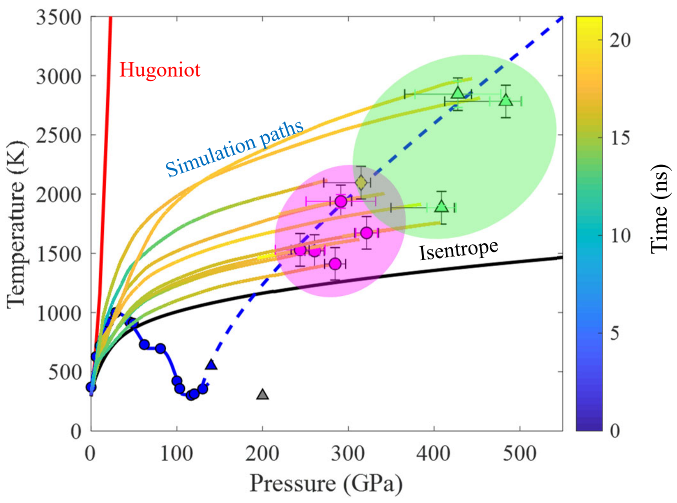

Supplementary Figure 7. **Ramp-compression paths.** The phase diagram of Na showing the paths (multi-color curves) of the experiments as a function of time (colorbar) from hydrodynamics simulations. The paths are consistent with a weak initial shock in the Na sample (<10 GPa). Our laser-driven ramp compression data are the pink circles, yellow diamond, and green triangles. The data are compared to the theoretical principal Hugoniot (*SESAME* 2441) and isentrope (*LEOS* 110). The melting curve data from Refs. [16, 17] are shown (blue circles and triangle) along with a Kechin [18] fit to density-functional-theory (DFT) calculations [19] for the melting curve (blue dashed line) above 130 GPa in the *hP4* phase. The highest pressure static compression data point from Ref. [20] is shown (gray triangle).

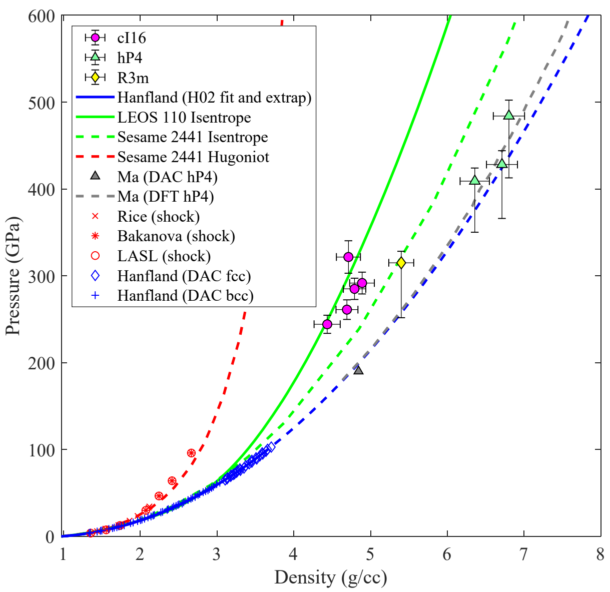

Supplementary Figure 8. **Pressure versus density.** Pressure versus density for the *hP4* phase (green triangles), *cI16* (pink circles), and $R\bar{3}m$ data (yellow diamond) compared to the DFT cold curve in the *hP4* phase (dashed-gray curve) [20], pressure-volume data and the *H02* relation fit and extrapolation measured by Hanfland *et al.* up to 100 GPa (blue points and blue curve; extrapolation blue-dashed curve)[21], *SESAME* 2441 principal isentrope (dashed green curve) and Hugoniot (dashed-red curve), LEOS 110 principal isentrope (green curve), and shock hugoniot data from Refs. [22–24].

cause a ∽50-J, 10-ns pulse and diamond ablators are used in nearly all experiments as shown in SI Figs. 1 and 2. The hydrodynamics simulation paths for each shot are shown in Supplementary Figure 7 and are consistent with a weak initial shock in the Na sample (< 10GPa). The error bars are given by the average range of temperatures spanned by simulation results within a factor of 2 of the minimum χ^2 difference between the measured and simulated velocities. No systematic errors in the temperature are taken into account. The effects of thermal conduction on the inferred temperature were investigated. The thermal conductivity used in the simulations is Spitzer/Braginski/Lee-More plasma thermal conductivity. The simulations suggest that the layers of the target do not thermalize on the timescales relevant to the experiment (∼20-ns) and the heat affected region of the sample is confined to a thin layer adjacent to the window. Simulations with and without the 0.1-μm Ti coating suggest that the Ti does not disturb the hydrodynamics and acts like a reflectance monitor as intended.

The temperature of ramp-compressed Na is inferred from HYADES hydrodynamics simulations and an experimental upper bound is given by streaked optical pyrometry (SOP) measurements [15]. The SOP counts are converted to temperature at the Na-LiF interface following the procedure of Gregor *et al.*. The Na-LiF interface temperature is inferred using calibration constants A0 = 288,600 ADU/ns, T0 = 1.909 eV, and a Na emissivity, ε = 1-R = 0.77, where R is the Na reflectivity. The reflectivity is constrained by the corresponding VISAR measurements at 532-nm where it was observed to drop to 23%±4% at the x-ray probe time. Based on experimental data on the refractive-index of ramp-compressed LiF to 900, LiF should be transparent at these conditions [7]. The LiF is assumed to be neither absorbing nor emitting over the 590-700 nm diagnostic wavelengths. The experimental data provides an upper bound on the temperature because the LiF is likely to have increasing absorption as the LiF is compressed and heated. This is likely confirmed by the observation that the reference Ti signal is observed to gradually decrease with time.

B. Structure determination

A summary of the data from this work and previous studies in shown in Supplementary Figure 8. The density of the *hP4* phase (green triangles), *cI16* (pink circles), and $R\bar{3}m$ data (yellow diamond) are calculated from the XRD data and compared to various theoretical and experimental data [20–24]. IP data and a comparison of the integrated diffraction pattern to the *hP4*, *cI16*, and $R\bar{3}m$ fit are shown in SI Figs. 9, 10, and 11.

To ensure the indexed diffraction peaks are from compressed Na, the diffraction patterns were compared to the expected diffraction patterns from the window materials, LiF and MgO, and the tungsten pinhole. Supplementary Informa-

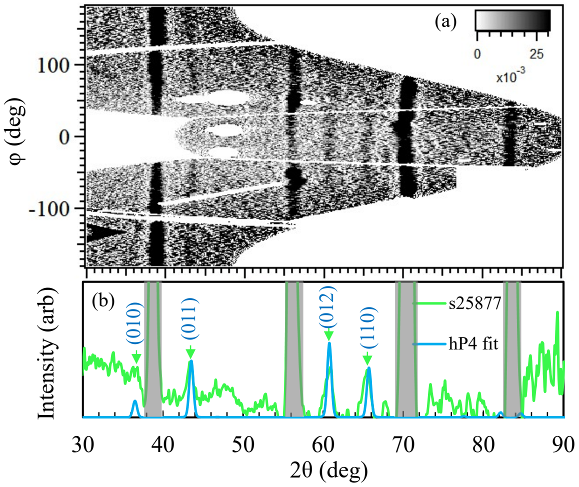

Supplementary Figure 9. ***hP4* x-ray diffraction pattern.** (a) Background subtracted 2θ-ϕ projections of a Na XRD pattern at 409±15 GPa (shot 25877). (b) The data are compared to the diffraction pattern from the *hP4* structure. The reflections from compressed Na are marked with green arrows. The gray shaded regions mark the calibration diffraction peaks.

tion Figure 12 shows a comparison of the diffraction data to the MgO *SESAME* 7640 and LiF *SESAME* 7271 v3 isentropes (black curves) and the ambient density tungsten *d*-spacings (dashed gray lines). Although the diffraction peak observed near 1.8 Å is consistent with the LiF (111) line, we believe this peak is from the compressed Na sample because the same peak is observed using an MgO window. In addition, previous experiments using single-crystal LiF as a diagnostic window have not observed a significant change in the LiF texture with compression. The data are also compared to the uncompressed bcc tungsten *d*-spacings because reference calibration diffraction lines from W are used in these experiments; in some cases, tantalum (bcc) is used instead. Although the reference material is chosen to minimize overlap with the compressed Na diffraction data, the pinhole is responsible the absence of the *cI16* (013) peak.

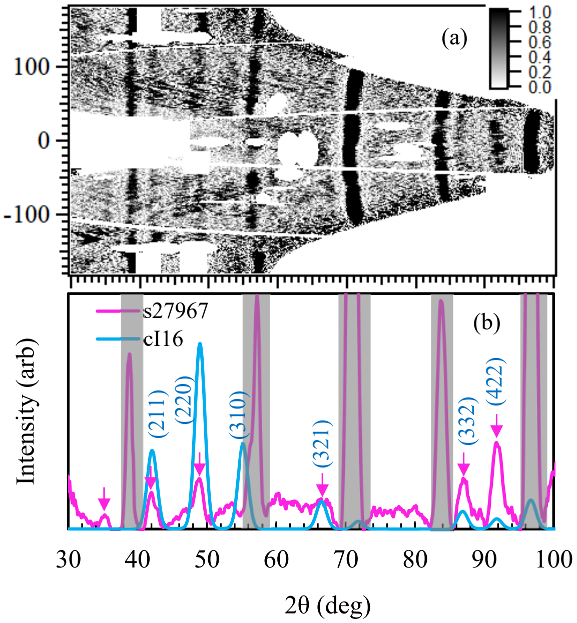

Supplementary Figure 10. ***cI16* x-ray diffraction pattern.** (a) Background subtracted 2θ-ϕ projections of a Na XRD pattern at 261±11 GPa (shot 27967). (b) The data are compared to the diffraction pattern from the *cI16* structure (space group: $I\bar{4}3d$, 220) with sixteen atoms on the *16c* Wyckoff site, (*x, x, x*) with a lattice parameter of a = 5.05 ± 0.05 Å and x = 0.043 (x = 0 for bcc). The reflections from compressed Na are marked with pink arrows. The gray shaded regions mark the calibration diffraction peaks.

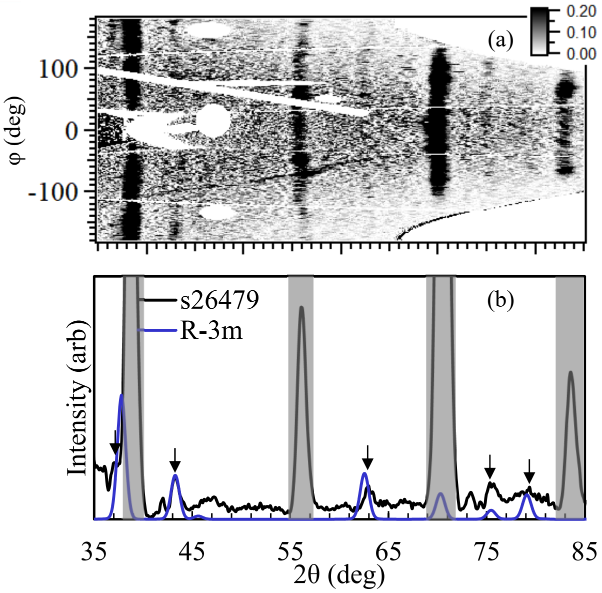

Supplementary Figure 11. **$R\bar{3}m$ x-ray diffraction pattern.** (a) Background subtracted 2θ-ϕ projections of a Na XRD pattern at 315±11 GPa (shot 26479). (b) The data are compared to the $R\bar{3}m$ structure with lattice parameters, $a = b$ = 2.85 ± 0.03 Å and c = 6.02 ± 0.06 Å (hexagonal axes) with atoms on the *6c* Wyckoff site, (*0, 0, z*), where z = 0.364. The reflections from compressed Na are marked with black arrows. The gray shaded regions mark the calibration diffraction peaks.

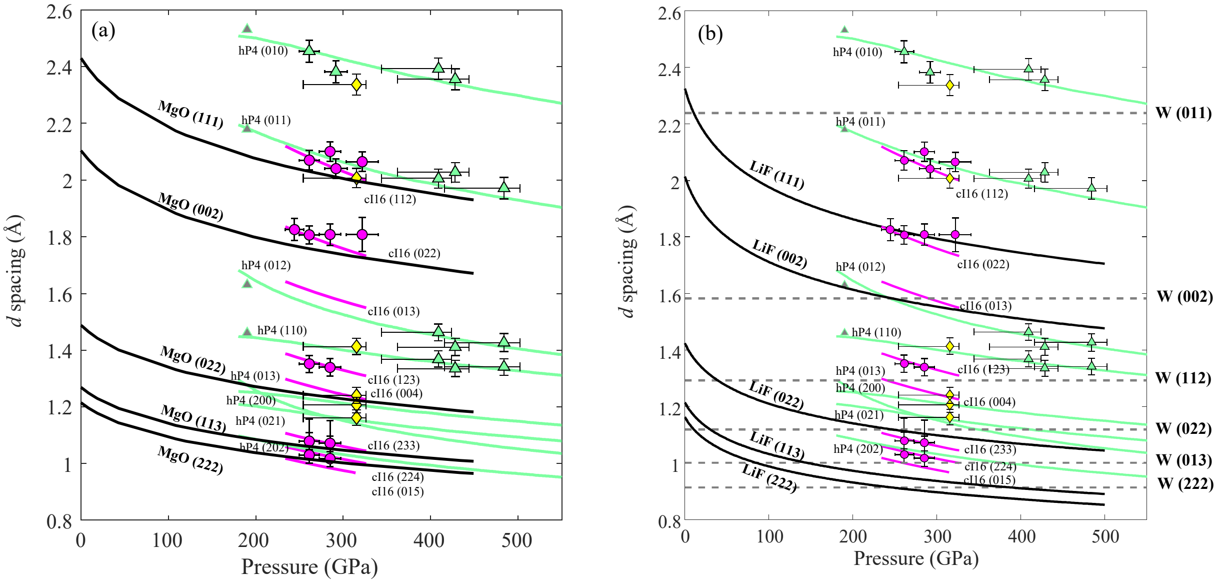

Supplementary Figure 12. **Comparison of the x-ray diffraction data to the x-ray diffraction from the window and pinhole materials.** (a) A comparison of the measured *d*-spacing versus pressure from this work (green triangles, pink circles, yellow diamonds), 0-K DFT EOS calculated by Ma *et al.* (green curves) assuming the *hP4* structure, the *hP4 d*-spacings calculated from the reported lattice parameters by Ma *et al.* at 190 GPa (gray triangles)[20], and the *SESAME* 7460 MgO principal isentrope (black curves). (b) The same data compared to the *SESAME* 7271 v3 LiF principal isentrope (black curves) and the ambient density tungsten *d*-spacings (dashed gray lines).

III. SUPPLEMENTARY TABLES

Supplementary Table I. Sodium diffraction data summary. The target dimension notation is Material [Thickness] and C is $\langle 110 \rangle$-oriented single-crystal diamond. All experiments used a 8.37-keV (Cu foil) x-ray energy. The probe times are the start of the 1-ns XRS pulse duration. The systematic asymmetric pressure error bar of -50 GPa is not included here. The error associated with the timing is ±58 ps including considerations in beam timing, absolute VISAR timing, etc.

Shot	Pressure (GPa)	Target Dimensions (μm)	2θ (deg)	Probe time (ns)	Density (g/cc)
26479	315 ± 11	C[26]Na[32]C[67]	36.96 ± 0.05<br>43.31 ± 0.2<br>63.22 ± 0.2<br>73.34 ± 0.20<br>75.72 ± 0.2<br>79.20 ± 0.2	17.96	5.4±0.2 ($R\bar{3}m$)
25877	409 ± 15	C[40]Na[46]C[77]	36.10 ± 0.2<br>43.34 ± 0.2<br>60.8 ± 0.2<br>65.54 ± 0.2	20.33	6.4±0.3 (*hP4*)
26480	428 ± 16	C[29]Na[65]C[71]	36.65 ± 0.15<br>42.84 ± 0.2<br>63.31 ± 0.2<br>67.37 ± 0.2	17.49	6.7±0.3 (*hP4*)
26475	484 ± 18	C[24]Na[55]C[71]	44.14 ± 0.3<br>62.55 ± 0.3<br>67.05 ± 0.2	18.19	6.8±0.3 (*hP4*)

Supplementary Table II. Simultaneous reflectivity and diffraction data summary. The target dimension notation is Material [Thickness] and C is $\langle 110 \rangle$-oriented single-crystal diamond, LiF is $\langle 100 \rangle$-oriented single-crystal LiF, and MgO is $\langle 100 \rangle$-oriented single-crystal MgO. All experiments used a 8.37-keV (Cu foil) x-ray energy. The probe times are the start of the 1-ns XRS pulse duration. The error associated with the timing is ±58 ps including considerations in beam timing, absolute VISAR timing, etc.

Shot	Pressure (GPa)	Target Dimensions (μm)	2θ (deg)	Probe time (ns)	Density (g/cc)
27794	244 ± 11	C[30]Na[31]LiF[103]	47.87 ± 0.4	19.21	4.4±0.2 (*cI16*)
27967	261 ± 11	C[25]Na[15]LiF[101]	35.12 ± 0.2<br>41.92 ± 0.2<br>48.40 ± 0.2<br>66.45 ± 0.3<br>86.73 ± 0.4<br>91.92 ± 0.4	17.93	4.7±0.1 (*cI16*)
27973	285 ± 13	C[28]Na[8]LiF[103]	41.28 ± 0.2<br>48.36 ± 0.4<br>67.15 ± 0.3<br>87.40 ± 0.4<br>93.43 ± 0.4	18.27	4.8±0.2 (*cI16*)
27790	292 ± 13	C[35]Na[33]LiF[102]	36.23 ± 0.2<br>42.56 ± 0.2	19.83	4.9 ± 0.2 (*cI16*)
27971	322 ± 14	C[28]Na[27]MgO[82.6]	42.04 ± 0.2<br>48.36 ± 1.0	19.36	4.7 ± 0.2 (*cI16*)

SUPPLMENTARY REFERENCES

[1] F. Coppari, R. Smith, D. Thorn, J. Rygg, D. Liedahl, R. Kraus, A. Lazicki, M. Millot, and J. Eggert, Optimized x-ray sources for x-ray diffraction measurements at the omega laser facility, Rev. Sci. Instrum. **90**, 125113 (2019).

[2] J. R. Rygg, J. H. Eggert, A. E. Lazicki, F. Coppari, J. A. Hawreliak, D. G. Hicks, R. F. Smith, C. M. Sorce, T. M. Uphaus, B. Yaakobi, *et al.*, Powder diffraction from solids in the terapascal regime, Rev. Sci. Instrum. **83**, 113904 (2012).

[3] J. Rygg, R. Smith, A. Lazicki, D. Braun, D. Fratanduono, R. Kraus, J. McNaney, D. Swift, C. Wehrenberg, F. Coppari, and *et al.*, X-ray diffraction at the national ignition facility, Rev. Sci. Instrum. **91**, 043902 (2020).

[4] P. M. Celliers, D. K. Bradley, G. W. Collins, D. G. Hicks, T. R. Boehly, and W. J. Armstrong, Line-imaging velocimeter for shock diagnostics at the omega laser facility, Rev. Sci. Instrum. **75**, 4916 (2004).

[5] S. Rothman and J. Maw, Characteristics analysis of isentropic compression experiments (ice), in *Journal de Physique IV (Proceedings)*, Vol. 134 (EDP sciences, 2006) pp. 745–750.

[6] J. T. Larsen and S. M. Lane, Hyades—a plasma hydrodynamics code for dense plasma studies, J. Quant. Spectrosc. Radiat. Transf. **51**, 179 (1994).

[7] L. E. Kirsch, S. J. Ali, D. E. Fratanduono, R. G. Kraus, D. G. Braun, A. Fernandez-Pañella, R. F. Smith, J. M. McNaney, and J. H. Eggert, Refractive index of lithium fluoride to 900 gigapascal and implications for dynamic equation of state measurements, J. Appl. Phys. **125**, 175901 (2019).

[8] G. Stevens, L. Veeser, P. Rigg, and R. Hixson, Suitability of magnesium oxide as a visar window, in *AIP Conf. Proc.*, Vol. 845 (American Institute of Physics, 2006) pp. 1353–1356.

[9] S. P. Lyon and J. D. Johnson, Sesame: the los alamos national laboratory equation of state database, Los Alamos National Laboratory, Los Alamos, NM, LA-UR-92-3407 (1992).

[10] D. Orlikowski, A. A. Correa, E. Schwegler, and J. E. Klepeis, A steinberg-guinan model for high-pressure carbon: Diamond phase, AIP Conference Proceedings **955**, 247 (2007).

[11] D. K. Bradley, J. H. Eggert, R. F. Smith, S. T. Prisbrey, D. G. Hicks, D. G. Braun, J. Biener, A. V. Hamza, R. E. Rudd, and G. W. Collins, Diamond at 800 gpa, Phys. Rev. Lett. **102**, 075503 (2009).

[12] J. K. Wicks, R. F. Smith, D. E. Fratanduono, F. Coppari, R. G. Kraus, M. G. Newman, J. R. Rygg, J. H. Eggert, and T. S. Duffy, Crystal structure and equation of state of fe-si alloys at super-earth core conditions, Sci. Adv. **4**, eaao5864 (2018).

[13] C. T. Seagle, J.-P. Davis, and M. D. Knudson, Mechanical response of lithium fluoride under off-principal dynamic shock-ramp loading, J. Appl. Phys. **120**, 165902 (2016).

[14] F. Coppari, R. Smith, J. Eggert, J. Wang, J. Rygg, A. Lazicki, J. Hawreliak, G. Collins, and T. Duffy, Experimental evidence for a phase transition in magnesium oxide at exoplanet pressures, Nature Geoscience **6**, 926 (2013).

[15] M. Gregor, R. Boni, A. Sorce, J. Kendrick, C. McCoy, D. Polsin, T. Boehly, P. Celliers, G. Collins, and D. Fratanduono, Absolute calibration of the omega streaked optical pyrometer for temperature measurements of compressed materials, Rev. Sci. Instrum. **87**, 114903 (2016).

[16] E. Gregoryanz, O. Degtyareva, M. Somayazulu, R. J. Hemley, and H.-k. Mao, Melting of dense sodium, Phys. Rev. Lett. **94**, 185502 (2005).

[17] M. Marqués, M. Santoro, C. L. Guillaume, F. A. Gorelli, J. Contreras-García, R. T. Howie, A. F. Goncharov, and E. Gregoryanz, Optical and electronic properties of dense sodium, Phys. Rev. B **83**, 184106 (2011).

[18] V. V. Kechin, Melting curve equations at high pressure, Phys. Rev. B. **65**, 052102 (2001).

[19] R. Paul, S. Hu, V. Karasiev, S. Bonev, and D. Polsin, Thermal effects on the electronic properties of sodium electride under high pressures, Phys. Rev. B **102**, 094103 (2020).

[20] Y. Ma, M. Eremets, A. R. Oganov, Y. Xie, I. Trojan, S. Medvedev, A. O. Lyakhov, M. Valle, and V. Prakapenka, Transparent dense sodium, Nature **458**, 182 (2009).

[21] M. Hanfland, I. Loa, and K. Syassen, Sodium under pressure: bcc to fcc structural transition and pressure-volume relation to 100 gpa, Phys. Rev. B. **65**, 184109 (2002).

[22] S. P. Marsh, Lasl shock hugoniot data.

[23] A. Bakanova, I. P. Dudoladov, and R. F. Trunin, Compression of alkali metals by strong shock waves, J Fiz. Tverd. Tela **7**, 1615 (1965).

[24] M. Rice, Pressure-volume relations for the alkali metals from shock-wave measurements, J. Phys. Chem. Solids **26**, 483 (1965).
